# Syndecan-1 as a severity biomarker for patients with trauma

**DOI:** 10.3389/fmed.2022.985955

**Published:** 2022-09-27

**Authors:** Keiko Suzuki, Hideshi Okada, Kazuyuki Sumi, Hiroyuki Tomita, Ryo Kobayashi, Takuma Ishihara, Yosuke Mizuno, Fuminori Yamaji, Ryo Kamidani, Tomotaka Miura, Ryu Yasuda, Yuichiro Kitagawa, Tetsuya Fukuta, Kodai Suzuki, Takahito Miyake, Norihide Kanda, Tomoaki Doi, Takahiro Yoshida, Shozo Yoshida, Nobuyuki Tetsuka, Shinji Ogura, Akio Suzuki

**Affiliations:** ^1^Department of Infection Control, Gifu University Graduate School of Medicine, Gifu, Japan; ^2^Department of Pharmacy, Gifu University Hospital, Gifu, Japan; ^3^Department of Emergency and Disaster Medicine, Gifu University Graduate School of Medicine, Gifu, Japan; ^4^Department of Tumor Pathology, Gifu University Graduate School of Medicine, Gifu, Japan; ^5^Laboratory of Advanced Medical Pharmacy, Gifu Pharmaceutical University, Gifu, Japan; ^6^Innovative and Clinical Research Promotion Center, Gifu University Hospital, Gifu, Japan; ^7^Department of Abuse Prevention Emergency Medicine, Gifu University Graduate School of Medicine, Gifu, Japan

**Keywords:** trauma, syndecan-1, injury severity score, glycocalyx, probability of survival

## Abstract

Tissue injury and hemorrhage induced by trauma lead to degradation of the endothelial glycocalyx, causing syndecan-1 (SDC-1) to be shed into the blood. In this study, we investigated whether serum SDC-1 is useful for evaluating trauma severity in patients. A single-center, retrospective, observational study was conducted at Gifu University Hospital. Patients transported to the emergency room for trauma and subsequently admitted to the intensive care unit from January 2019 to December 2021 were enrolled. A linear regression model was constructed to evaluate the association of serum SDC-1 with injury severity score (ISS) and probability of survival (Ps). A total of 76 trauma patients (54 men and 22 women) were analyzed. ISS was significantly associated with serum SDC-1 level in trauma patients. Among the six body regions defined in the AIS used to calculate the ISS score, “chest” and “abdominal or pelvic contents” were significantly associated with serum SDC-1 level, and “extremities or pelvic girdle” also tended to show an association with serum SDC-1 level. Moreover, increasing serum SDC-1 level was significantly correlated with decreasing Ps. Serum SDC-1 may be a useful biomarker for monitoring the severity of trauma in patients. Further large-scale studies are warranted to verify these results.

## Introduction

Trauma is a global phenomenon and one of the main causes of death worldwide (1). The major causes of trauma-related death within the first 24 h are hemorrhage and brain injury, and respiratory distress, organ failure, and infection in the period thereafter (2). Because trauma has a wide range of presentations, both in injury type and the location and effect of individual wounds, the ability to measure injury severity is particularly important for comparison of disparate types of trauma. The Injury Severity Score (ISS) is an anatomical scoring system that provides an overall score for patients with multiple injuries, in which each injury is assigned an Abbreviated Injury Scale (AIS) score (3, 4). While the ISS is regarded as the gold standard for grading trauma severity (5, 6), it is important to note that trauma severity measured using the ISS is anatomically based, and reveals no information on disease pathophysiology. However, the systemic response induced by tissue injury and hemorrhage, which includes the release of damage-associated molecular patterns (DAMPs), hypoperfusion and reperfusion, inflammatory responses, and activation of endocrine and neurological systems, leads to endothelial cell activation, resulting in cellular and organ dysfunction (6). Thus, although the pathophysiology of trauma is characterized by endothelial injury, this is not considered by the ISS.

The endothelial glycocalyx, a gel-like layer of glycoproteins that covers the luminal surface of the capillary endothelium, is thought to maintain organ and vascular homeostasis (7). Syndecan-1 (SDC-1), the core protein in heparan sulfate proteoglycan, is found in the endothelial glycocalyx and shed into the blood in various systemic inflammatory conditions, including trauma (7, 8), sepsis (9), acute respiratory distress syndrome (10), acute kidney injury (11) and cardiovascular disease (12, 13). Additionally, several studies have demonstrated an association between serum SDC-1 level and mortality in trauma patients (14, 15). A prospective cohort study of 75 trauma patients reported that serum SDC-1 level ≥ 63 ng/ml was an independent factor for 30-day mortality after adjusting for age and ISS (15). Similarly, a prospective observational study of 410 trauma patients revealed that serum SDC-1 level ≥ 40 ng/ml was a significant factor for 30-day in-hospital mortality using multivariable logistic analysis adjusted for age, ISS, arrival systolic blood pressure, and base excess (14). However, it is unclear whether serum SDC-1 is a useful marker of severity in patients with trauma.

The aim of this study was to investigate the relationship between ISS and serum SDC-1 levels on admission to the emergency room in trauma patients.

## Methods

### Patients

This single-center retrospective observational study was conducted at Gifu University Hospital, which is affiliated with Gifu University (Gifu, Japan). Patients transported to the emergency room (ER) for trauma and admitted to the intensive care unit at Gifu University from January 2019 to December 2021 were included. Patients were excluded from the present analysis if they were transported to hospital 24 h or more after the onset of injury, had an unclear onset time of injury, were diagnosed with an AIS of < 3, received cardiopulmonary resuscitation or fluid resuscitation during transport, were undergoing maintenance dialysis, or were aged under 20 years.

### Data collection and study design

Upon transport to the ER, blood was sampled from eligible patients. Data derived from these blood samples were used in the present analysis. All laboratory data, except serum SDC-1, and patient demographics were extracted from the hospital's electronic medical records.

AIS was scored by physicians using AIS2008 (16). To calculate the ISS score, we used the six body regions defined in the AIS: 1. Head and neck, 2. Face, 3. Chest, 4. Abdomen, 5. Extremities pelvis, 6. Surface. The ISS score was calculated from the three most severely injured of the six body regions using the equation: ISS = (AIS_1_)^2^ ± (AIS_2_)^2^ ± (AIS_3_)^2^.

The probability of survival (Ps) is a commonly used parameter in the field of trauma care. It can be calculated from the AIS, ISS and Trauma and Injury Severity Score (TRISS) as follows:


$$\begin{array}{lll} Ps\; & = & \;1/\left(1\pm \;{\text{e}}^{-\text{b}}\right)\\ \text{e} & = & 2.718282\;\left(base\;of\;natural\;logarithm\right)\\ \text{b} & = & {\text{b}}_{0}\pm {\text{b}}_{1}\left(\text{RTS}\right)\pm {\text{b}}_{2}\left(\text{ISS}\right)\pm {\text{b}}_{3}\left(\text{AgeIndex}\right) \end{array}$$

where RTS is the revised trauma score and *b*_0_, *b*_1_, *b*_2_, *b*_3_ are coefficients. The coefficients b0–b3 are derived from multiple-regression analysis of the Major Trauma Outcome Study database (17). For patients under 55 years old, the age index is 0, while for patients > 55 years old, the age index is 1. These coefficients change depending on whether subjects are pediatric patients or have blunt trauma. RTS can be calculated from the following three physiological parameters:


$$\begin{array}{l} \text{RTS }=\;0.9368\;\left(\text{GCS}\right)\pm 0.7326\;\left(\text{SBP}\right)\pm 0.2908\;\left(\text{RR}\right) \end{array}$$

where GCS is the Glasgow Coma Scale, SBP is systolic blood pressure (mmHg), and RR is respiratory rate (/min). Serum SDC-1 concentrations were measured using an enzyme-linked immunosorbent assay (950.640.192; Diaclone, Besancon, Cedex, France). These data were retrospectively analyzed.

### Statistical analysis

Patients' baseline characteristics are presented as median and interquartile range (IQR) for continuous variables, and frequency and proportion for categorical variables. To evaluate the association between ISS and serum SDC-1, we performed multivariable regression analysis adjusted for covariates we defined a priori based on factors previously shown to be strongly associated with serum SDC-1 and ISS. For serum SDC-1, these were age (18, 19), gender (20, 21), previous treatment with transfusion and/or angiography at another hospital before being transported to our hospital (22). For ISS, these were: medication history of antiplatelet use and/or anticoagulant use (23, 24). Because the distribution of serum SDC-1 was skewed, natural log transformation was used in the regression model and the results are presented with back-transformation.

To evaluate which of the six body regions defined in the AIS used to calculate the ISS score was associated with serum SDC-1, a similar analysis was performed by replacing the ISS with the body region-specific score. The association between Ps and serum SDC-1 was also evaluated using a multivariable linear regression model. A two-sided *P*-value < 0.05 was considered significant. All analyses were performed using R 4.1.1 (The R Project for Statistical Computing).

### Ethics statement

The investigation conformed with the principles outlined in the Declaration of Helsinki. Ethics approval was obtained from the medical ethics committee of Gifu University Graduate School of Medicine, Gifu, Japan (Institutional review board approval No. 2021-B097). In view of the retrospective nature of the study, subject informed consent was not required.

## Results

### Patient characteristics

**Figure 1:**
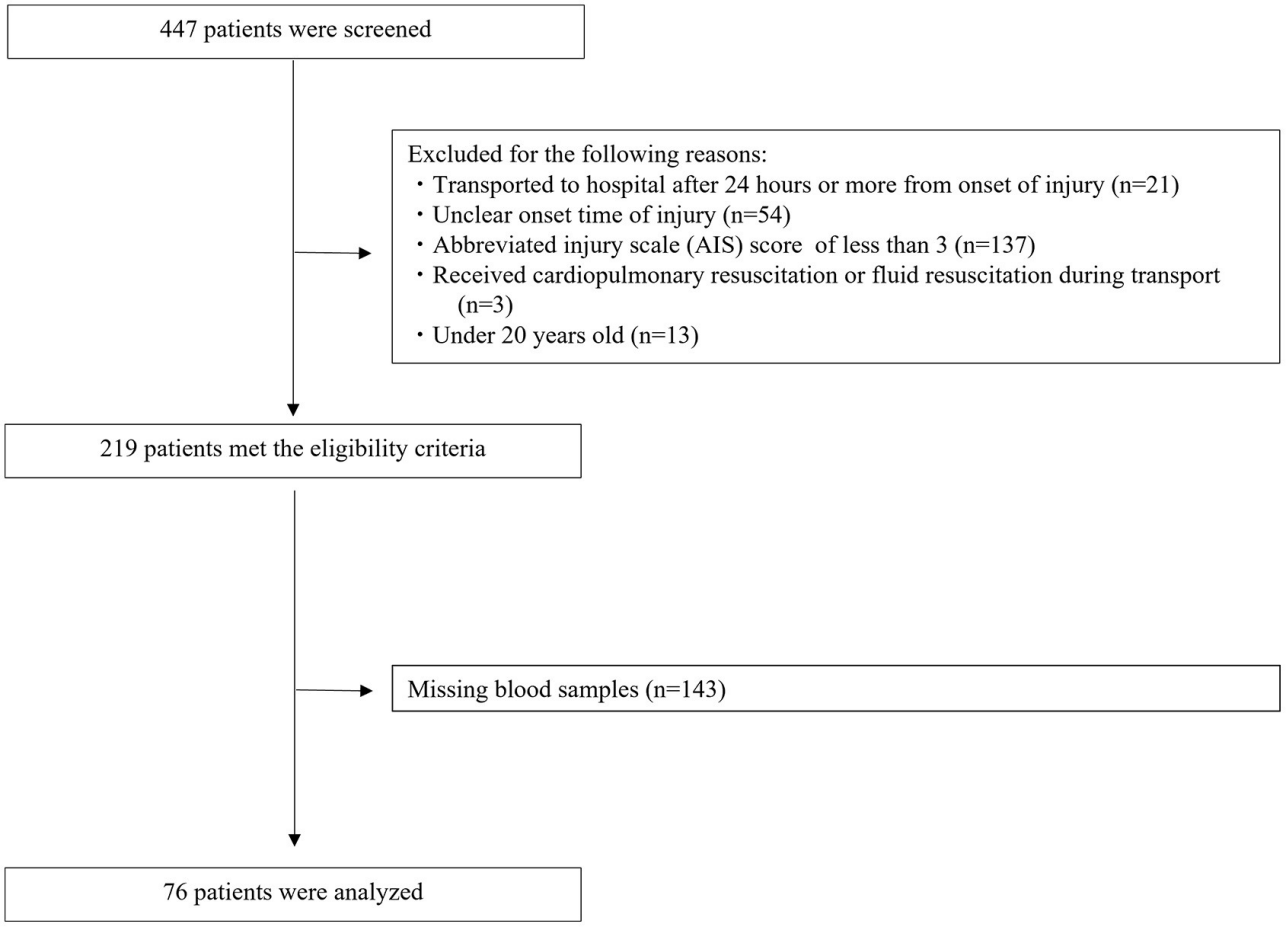
CONSORT diagram.

The disposition of the enrolled patients is shown in Figure 1. A total of 447 patients with trauma were transferred to our ER during the study period. Of these, 228 patients were excluded for the following reasons: AIS < 3 (*n* = 137), unclear onset time of injury (*n* = 54), transported to hospital 24 h or more after onset of injury (*n* = 21), under 20 years old (*n* = 13), and received cardiopulmonary resuscitation or fluid resuscitation during transport (*n* = 3). A total of 219 patients met the eligibility criteria. However, 143 of these patients had missing blood samples, which meant we could not determine their serum SDC-1 levels. Thus, the remaining 76 patients (54 men and 22 women) were analyzed in this study.

Patient demographics are shown in Table 1. Median age was 66.0 years (IQR, 47.0–78.0) and median duration of hospital stay was 29.0 days (IQR, 20.0–48.8). The most common cause of trauma was falling from a height (*n* = 35, 46.0%), followed by motor vehicle accident (*n* = 33, 43.4%) and others (*n* = 8, 10.5%). A total of 3 (3.9%) and 5 (6.6%) patients had received transfusion and angiography at another hospital before being transported to our hospital, respectively. Further, 6 (7.9%) and 3 (3.9%) patients were taking antiplatelet agents and anticoagulants, respectively.

**Table 1:** Patient demographics.

Age, years, median (IQR)	66.0 (47.0–78.0)
Sex, male/female, ***n*** (%)	54 (71.1)/22 (28.9)
**Treatment by previous doctor**, ***n*** **(%)**	
Transfusion	3 (3.9)
Angiography	5 (6.6)
**Medication**	
Antiplatelet agents	6 (7.9)
Anticoagulants	3 (3.9)
**Reasons for injury**, ***n*** **(%)**	
Falling accident	35 (46.0)
Traffic accident	33 (43.4)
Other	8 (10.5)
Length of hospital stay, days, median (IQR)	29.0 (20.0–48.8)
**Laboratory data on arrival at ER**	
Alb	3.7 (3.3–4.1)
AST	43.0 (28.8–77.0)
ALT	30.5 (20.8–50.5)
CRE	0.9 (0.7–1.1)
BUN	17.9 (13.8–21.6)
WBC	13.0 (8.2–16.9)
HGB	12.4 (10.3–13.9)
PLT	210.5 (161.5–255.0)
FIB	216.0 (187.5–279.8)
FDP	95.2 (29.5–226.9)
D-dimer	37.4 (12.1–75.7)

All data indicate median, 25–75th percentile unless otherwise indicated.

IQR, interquartile range; ER, emergency room; Alb, albumin; AST, aspartate aminotransferase; ALT, alanine aminotransferase; CRE, creatinine; BUN, blood urea nitrogen; WBC, white blood cells; HGB, hemoglobin, PLT; platelets, FIB; fibrinogen, FDP; fibrin degradation product.

### Relationship between ISS and serum SDC-1 levels on admission to the emergency room

**Figure 2:**
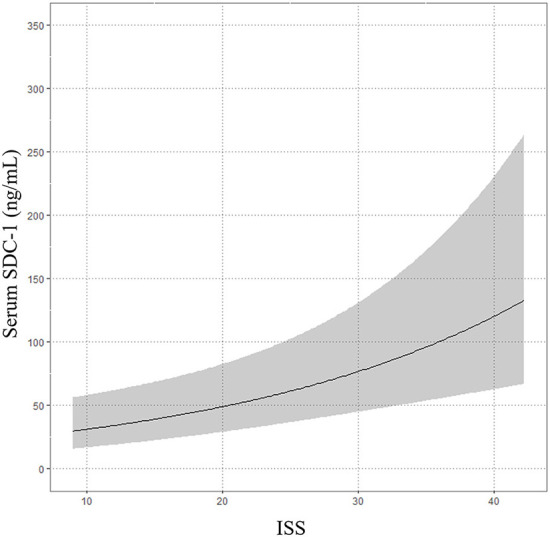
Association between serum SDC-1 and injury severity score (ISS).

The median time from onset of trauma to blood collection in the ER was 62.5 min (IQR, 39.75–150.25). The median serum SDC-1 level was 34.6 ng/mL (IQR, 27.1–67.8), and the median ISS score was 20 (IQR 13.8–29.0). The results of the multivariable linear regression model adjusted for age, gender, treatment history (transfusion and angiography) and medication history (antiplatelet agents and anticoagulants) are shown in Table 2 and Figure 2. ISS was significantly associated with serum SDC-1 level in trauma patients with AIS ≥3 [Exp (β) = 1.046, 95% confidence interval (CI) = 1.020–1.072, *P* = 0.001, *R*^2^ = 0.23].

**Table 2:** Linear regression between serum SDC-1 and ISS in trauma patients at arrival at the ER.

**Factor**	**Exp (β)**	**95%LCI**	**95%UCI**	***P*-value**	***R^2^***
ISS	1.046	1.02	1.072	0.001	0.23

Exp (β), exponentiation of the β coefficient; R^2^, coefficient of determination; IQR, interquartile range; LCI, lower confidence interval; UCI, upper confidence interval; ISS, injury severity score; ER, emergency room.

Adjusted for age, gender, previous treatment, and medication history (antiplatelets or anticoagulants).

**Figure 3:**
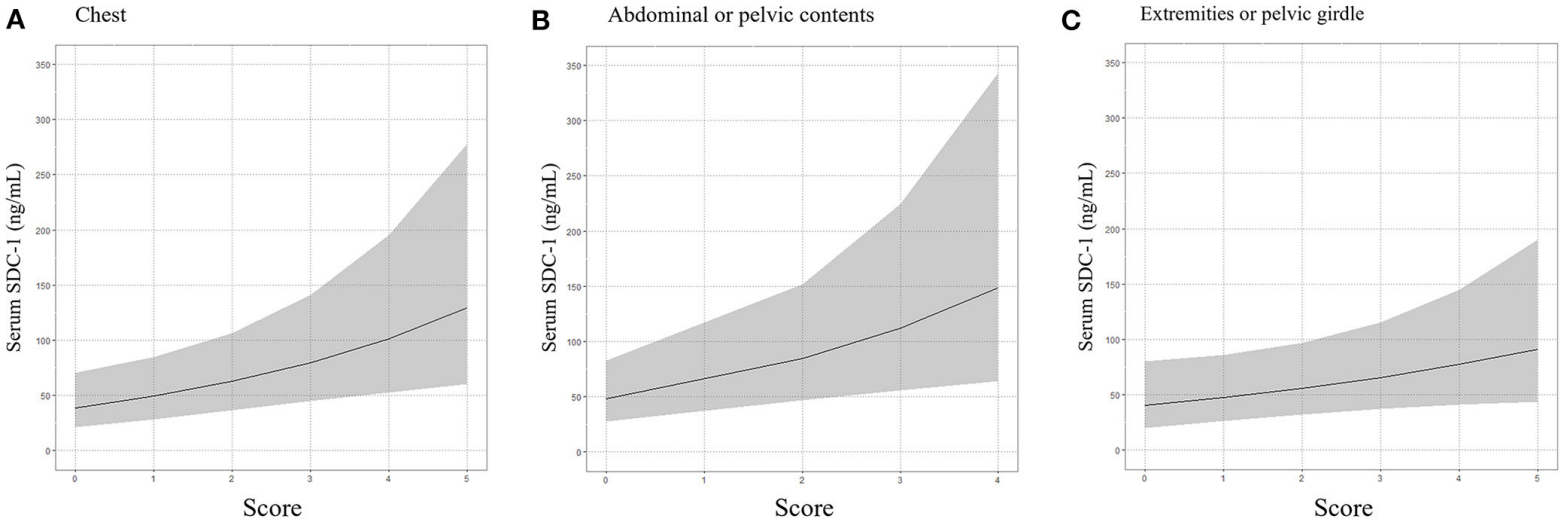
Association between serum SDC-1 and chest **(A)**, abdominal or pelvic contents **(B)** and extremities or pelvic girdle **(C)**, body regions defined by the AIS used to calculate ISS score.

We subsequently used a multivariable linear regression model adjusted for age, gender, treatment history (transfusion and angiography) and medication history (antiplatelet agents and anticoagulants) to determine whether the ISS score for each of the six body regions defined in the AIS was associated with serum SDC-1 level. As shown in Table 3 and Figure 3, the body region-specific score for “chest” and “abdominal or pelvic contents” were significantly associated with serum SDC-1 level in trauma patients (chest: Exp (β) = 1.273, 95%CI = 1.076–1.506, *P* = 0.006, *R*^2^ = 0.18; abdominal or pelvic contents: Exp (β) = 1.326, 95%CI = 1.090–1.614, *P* = 0.005, *R*^2^ = 0.18), and the score for “extremities or pelvic girdle” also tended to show an association with serum SDC-1 level (Exp (β) = 1.178, 95%CI = 0.982–1.412, *P* = 0.077, *R*^2^ = 0.12).

**Table 3:** Linear regression between serum SDC-1 and six body regions defined in the AIS used to calculate the ISS score in trauma patients on arrival at the ER.

**Body region**	**Exp (β)**	**95%LCI**	**95%UCI**	***P*-value**	***R* ^2^**
Head or neck	1.061	0.909	1.239	0.445	0.09
Face	1.073	0.736	1.566	0.71	0.08
Chest	1.273	1.076	1.506	0.006	0.18
Abdominal or pelvic contents	1.326	1.09	1.614	0.005	0.18
Extremities or pelvic girdle	1.178	0.982	1.412	0.077	0.12
External	0.876	0.467	1.643	0.676	0.08

Exp (β), exponentiation of the β coefficient, R^2^, coefficient of determination; IQR, interquartile range; LCI, lower confidence interval; UCI, upper confidence interval; ISS, injury severity score; ER, emergency room.

Adjusted for age, gender, previous treatment, and medication history (antiplatelets or anticoagulants).

### Relationship between probability of survival and serum SDC-1 level on admission to the emergency room

**Figure 4:**
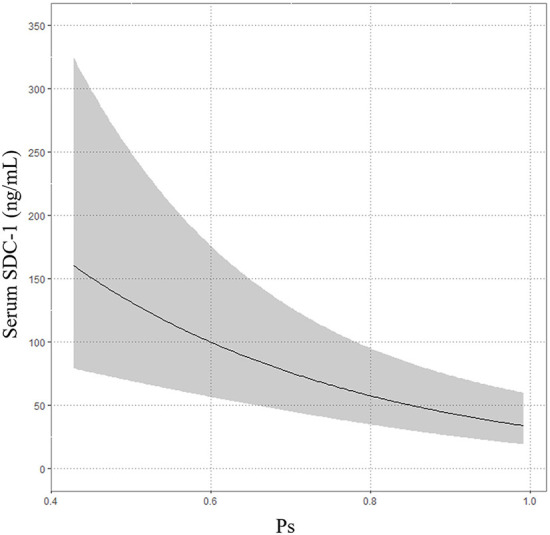
Association between serum SDC-1 and probability of survival (Ps).

The median Ps was 0.9225 (IQR 0.8134–0.9655). The multivariable linear regression model showed that increasing serum SDC-1 level was significantly correlated with decreasing Ps after adjustment for age, gender, treatment history (transfusion and angiography) and medication history (antiplatelet agents and anticoagulants) (Exp (β) = 0.973, 95%CI = 0.96–0.986, *P* < 0.001, *R*^2^ = 0.26, Table 4; Figure 4).

**Table 4:** Linear regression between serum SDC-1 and probability of survival in trauma patients at arrival at the ER.

**Factor**	Exp (β) *	**95%LCI**	**95%UCI**	***P*-value**	***R^2^***
Ps (/0.01)	0.973	0.96	0.986	< 0.001	0.26

Exp (β), exponentiation of the β coefficient; R^2^, coefficient of determination; IQR, interquartile range; LCI, lower confidence interval; UCI, upper confidence interval; Ps, probability of survival; ER, emergency room.

Adjusted for age, gender, previous treatment, and medication history (antiplatelets or anticoagulants).

*The value for increments of 0.01 in Ps.

## Discussion

We demonstrated that serum SDC-1 level measured on admission to the emergency room in patients with trauma was significantly associated with ISS. Among the six body regions defined in the AIS used to calculate the ISS score, “chest” and “abdominal or pelvic contents” were significantly associated with serum SDC-1 level in trauma patients, and “extremities or pelvic girdle” tended to show an association with serum SDC-1 level. Moreover, increasing serum SDC-1 level was significantly correlated with decreasing Ps.

The composition and function of the endothelial glycocalyx are related to the pathophysiology of trauma (8), with the shedding of SDC-1 from the endothelial glycocalyx into serum having been shown to be associated with several inflammatory cytokines and chemical mediators related to the pathophysiology of trauma (14, 15, 25). The ISS is correlated with adrenaline, interleukin (IL)-6, histone-complexed DNA fragments, high-mobility group box 1 (HMGB1), soluble thrombomodulin (sTM) and protein C in trauma patients with high serum SDC-1 levels (≥ 63 ng/ml) but not in those with low SDC-1 levels (< 63 ng/ml) (15). Patients with serum SDC-1 ≥ 40 ng/ml suffering from blunt or penetrating trauma have nearly 1.5-fold higher sTM levels in the initial 24 h after the trauma than patients with serum SDC-1 < 40 ng/ml (14). Serum SDC-1 levels in severely injured patients experiencing hemorrhagic shock are positively correlated with interleukin-10 and inversely correlated with interferon (IF)-γ, fractalkine and IL-1β (25). These findings suggest that serum SDC-1 may reflect the pathophysiology of trauma.

Two clinical studies in trauma patients have also reported relationships between serum SDC-1 and ISS. In an observational study of 80 trauma patients, Johansson et al. showed that those with acute coagulopathy of trauma shock (ACoTS), defined as activated partial thromboplastin time or international normalized ratio above the normal reference level, had significantly higher median ISS values and serum SDC-1 levels than non-ACoTS patients [ISS: 34 (IQR 30–43) vs. 17 (IQR 10–25), *P* < 0.001; SDC-1: 62 (IQR 34–107) *vs* 31 (IQR 18–48), *P* = 0.013] (26). The same group also reported in a prospective cohort study of 75 trauma patients that the ISS did not significantly differ between a high serum SDC-1 group and low serum SDC-1 group (15). However, it is important to note that the median ISS was higher in the high serum SDC-1 group than in the low serum SDC-1 group [23 (IQR 14–37) vs. 17 (IQR 14–28)]. Additionally, neither of the two studies described above used continuous values to analyze the relationship between SDC-1 level and ISS, nor did they discuss the validity of grouping by the median value. In fact, the median serum SDC-1 level and ISS score in the present study was different to those of the prior studies. In the present study, multivariable regression analysis adjusted for age, gender, previous treatment, and medication history demonstrated that serum SDC-1 level measured on admission to the emergency room in trauma patients was significantly associated with ISS. This result indicates that serum SDC-1 may be a useful biomarker of severity in patients with trauma.

Moreover, we showed that among the six body regions defined in the AIS used to calculate the ISS score, “chest” and “abdominal or pelvic contents” were significantly associated with serum SDC-1 level, and that “extremities or pelvic girdle” tended to be associated with serum SDC-1 level. In contrast, “head or neck,” “face” and “external” regions were not associated with serum SDC-1 level. The reason for the difference in association between serum SDC-1 and the ISS score for each body region defined by the AIS is unclear. However, we speculate that the total surface area of the endothelium in each region may differentially influence the amount of serum SDC-1. For example, the “head and neck,” “face” and “external” regions, mainly categorized as ectodermic tissue, have a small surface area of endothelium, whereas “chest” and “abdominal or pelvic contents” and “extremities or pelvic girdle,” mainly categorized as endodermic or mesodermic tissue, have a greater surface area of endothelium. Reports from surgeries of the abdominal, cardiac and thoracic regions have indicated elevated levels of serum SDC-1, which is consistent with the present results (27–29). Additionally, the structure and components of the endothelial glycocalyx differ among organs, as does its susceptibility to injury (7, 30). The endothelial glycocalyx in the capillaries of the brain, for example, unlike that in the capillaries of the heart and lungs, is extremely thick. As a result, lipopolysaccharide-induced vascular injury completely eliminates the endothelial glycocalyx in cardiac and pulmonary capillaries but leaves much of it in the cerebral capillaries intact (30). These factors may explain the difference in correlation between SDC-1 and the ISS score for some body regions defined by the AIS but not others.

In the present study, we used probability of survival to evaluate the association between serum SDC-1 level and mortality in trauma patients because mortality was only 3.947% (3/76). In the multivariable linear regression model, increasing serum SDC-1 level was significantly correlated with decreasing Ps, a result that is consistent with those of previous reports (15, 25).

This study has several limitations. First, we were unable to evaluate the extent of endothelium injury and serum inflammatory cytokine and chemical mediator levels (e.g., IL-6, histone-complexed DNA fragments, HMGB1, sTM, protein C) related to the pathophysiology of trauma. Second, in addition to the endothelial glycocalyx, SDC-1 is also expressed in other organs. However, we did not evaluate SDC-1 expression in different organs in this study. Third, we evaluated the Ps but not mortality in this study. Fourth, due to the retrospective nature of this study, a large number of patients (*n* = 143) who met the eligibility criteria were excluded because of missing blood samples, as this prevented us from determining their serum SDC-1 levels. Moreover, potentially relevant confounding factors may have been missed. Finally, the sample size was small and data were obtained from a single institution.

In conclusion, serum SDC-1 may be a useful biomarker for evaluating severity in trauma patients, especially trauma in the “chest,” “abdominal or pelvic contents” and “extremities or pelvic girdle” regions. Additionally, elevated serum SDC-1 levels may be an important risk factor for mortality in patients with trauma. Further large-scale studies are warranted to verify the usefulness of serum SDC-1 as a marker of severity in trauma patients.

## Data availability statement

The raw data supporting the conclusions of this article will be made available by the authors, without undue reservation.

## Ethics statement

The studies involving human participants were reviewed and approved by ethics approval was obtained from the Medical Ethics Committee of the Gifu University Graduate School of Medicine, Gifu, Japan (record no: 2021-B097). Written informed consent for participation was not required for this study in accordance with the national legislation and the institutional requirements.

## Author contributions

KeS and AS wrote the manuscript. KeS, KaS, YM, FY, RKo, TMiu, RY, YK, TF, KoS, TMiy, and NK collected the blood samples. KeS, KaS, and RKo measured the syndecan-1 concentration using ELISA. KeS and RY created the database. TI performed the statistical analysis. YM, FY, RKa, TMiu, RY, YK, TF, KoS, TMiy, NK, TD, TY, and SY treated the patients. HT, SY, NT, and SO supervised the study. HO and AS revised and edited the manuscript. All authors contributed to the article and approved the submitted version.

## Conflict of interest

The authors declare that the research was conducted in the absence of any commercial or financial relationships that could be construed as a potential conflict of interest.

## Publisher's note

All claims expressed in this article are solely those of the authors and do not necessarily represent those of their affiliated organizations, or those of the publisher, the editors and the reviewers. Any product that may be evaluated in this article, or claim that may be made by its manufacturer, is not guaranteed or endorsed by the publisher.
